# A framework to infer *de novo* exonic variants when parental genotypes are missing enhances association studies of autism

**DOI:** 10.1093/bioinformatics/btag177

**Published:** 2026-05-04

**Authors:** Haeun Moon, Laura Sloofman, Marina Natividad Avila, Lambertus Klei, Bernie Devlin, Joseph D Buxbaum, Kathryn Roeder

**Affiliations:** Department of Statistics, Seoul National University, Seoul, 08826, South Korea; School of Transdisciplinary Innovations, Seoul National University, Seoul, 08826, South Korea; Institute for Data Innovation in Science, Seoul National University, Seoul, 08826, South Korea; Seaver Autism Center for Research and Treatment, Icahn School of Medicine at Mount Sinai, New York, NY 10029, United States; Department of Psychiatry, Icahn School of Medicine at Mount Sinai, New York, NY 10029, United States; Department of Genetics and Genomic Sciences, Icahn School of Medicine at Mount Sinai, New York, NY 10029, United States; Friedman Brain Institute, Icahn School of Medicine at Mount Sinai, New York, NY 10029, United States; Department of Neuroscience, Icahn School of Medicine at Mount Sinai, New York, NY 10029, United States; The Mindich Child Health and Development Institute, Icahn School of Medicine at Mount Sinai, New York, NY 10029, United States; Seaver Autism Center for Research and Treatment, Icahn School of Medicine at Mount Sinai, New York, NY 10029, United States; Department of Psychiatry, Icahn School of Medicine at Mount Sinai, New York, NY 10029, United States; Department of Genetics and Genomic Sciences, Icahn School of Medicine at Mount Sinai, New York, NY 10029, United States; Friedman Brain Institute, Icahn School of Medicine at Mount Sinai, New York, NY 10029, United States; Department of Neuroscience, Icahn School of Medicine at Mount Sinai, New York, NY 10029, United States; The Mindich Child Health and Development Institute, Icahn School of Medicine at Mount Sinai, New York, NY 10029, United States; Department of Psychiatry, University of Pittsburgh, Pittsburgh, PA 15260, United States; Department of Psychiatry, University of Pittsburgh, Pittsburgh, PA 15260, United States; Seaver Autism Center for Research and Treatment, Icahn School of Medicine at Mount Sinai, New York, NY 10029, United States; Department of Psychiatry, Icahn School of Medicine at Mount Sinai, New York, NY 10029, United States; Department of Genetics and Genomic Sciences, Icahn School of Medicine at Mount Sinai, New York, NY 10029, United States; Friedman Brain Institute, Icahn School of Medicine at Mount Sinai, New York, NY 10029, United States; Department of Neuroscience, Icahn School of Medicine at Mount Sinai, New York, NY 10029, United States; The Mindich Child Health and Development Institute, Icahn School of Medicine at Mount Sinai, New York, NY 10029, United States; Department of Statistics and Data Science, Carnegie Mellon University, Pittsburgh, PA 15213, United States; Computational Biology Department, Carnegie Mellon University, Pittsburgh, PA 15213, United States

## Abstract

**Motivation:**

Gene-damaging mutations are highly informative for studies seeking to discover genes underlying developmental disorders. Traditionally, these *de novo* variants are recognized by evaluating high-quality DNA sequence from affected offspring and parents. However, when parental sequence is unavailable, methods are required to infer *de novo* status and use this inference for association studies.

**Results:**

We use data from autism spectrum disorder to illustrate and evaluate methods. Separating *de novo* from rare inherited variants is challenging because the latter are far more common. Using a classifier for unbalanced data and variants of known inheritance class, we build an inheritance model and then a *de novo* score for variants when parental data are missing. Next, we propose a new Random Draw (RD) model to use this score for gene discovery. Built into an existing inferential framework, RD produces a more powerful gene-based association test and controls the false discovery rate.

**Availability and implementation:**

Codes are available at Github (https://github.com/HaeunM/TADA-RD) and Zenodo (DOI: https://doi.org/10.5281/zenodo.18531769).

## Introduction

Rare genetic variation has been key in the identification of specific genes that alter development, including genes underlying congenital heart disease (Jin et al. 2017, Watkins et al. 2019) and autism spectrum disorder (Fu et al. 2022). For ASD, most of the information inherent in rare variation comes from *de novo* exonic mutations (Fu et al. 2022), and the same is true for chromatin modifiers underlying some forms of congenital heart disease (Jin et al. 2017). This presents challenges for study design. To identify a variant as *de novo*, definitively, requires high-quality whole-exome or whole-genome sequence data from an affected offspring (the proband) and both parents. An alternative design contrasts probands with appropriately matched controls. While the case-control design contributes some evidence for association, the information gleaned from rare variants in such a cohort is typically modest (Fu et al. 2022), in large part because which variants are *de novo* and which are inherited is hidden. We propose methods to recover this information.

This problem is challenging because there are far more inherited than *de novo* variants per subject. Consider autism spectrum disorder (ASD) as an example. Defining a rare variant as having a minor allele frequency MAF<0.001, a typical person diagnosed with ASD carries one to two *de novo* variants in their exons, compared to approximately thirty times more rare inherited variants (Fu et al. 2022). All classification problems are challenged in this setting. In addition, the classification of harmful from benign variants remains prone to errors, although *de novo* variants that affect liability must alter gene function and neurodevelopment. Furthermore, because only about 5% of the coding genes have a strong impact on ASD (He et al. 2013), and many of them are not yet known, not all *de novo* variation matters. Any model to infer *de novo* variation will benefit from information about association for specific genes.

For the problem of sorting *de novo* from inherited variants, frequencies of variants carry information. If a putative *de novo* variant is relatively common in the population, it is more likely to be an inherited variant that went undetected in the parents due to randomness of the sequencing process. Using this logic, we can reasonably expect that most true *de novo* variants are rare in the sample under study and rare or absent in other population databases. In addition, genes in which functional variation is subjected to evolutionary negative selection (Weghorn et al. 2019, Agarwal et al. 2023), constrained genes, are important for typical development, ASD and many other developmental disorders. A useful measure of gene constraint is LOEUF. Genes with low LOEUF scores are constrained (Karczewski et al. 2020): in a population, they tend to have relatively few variants, leading to loss-of-function of the gene. These loss-of-function variants, often annotated as protein-truncating variants or PTVs, are not the only kind of variation that can cause loss-of-function. However, in this work, we will focus on PTVs because they make up the majority of variants found in ASD subjects, developing a classifier to separate *de novo* from inherited PTVs.

**Figure 1:**
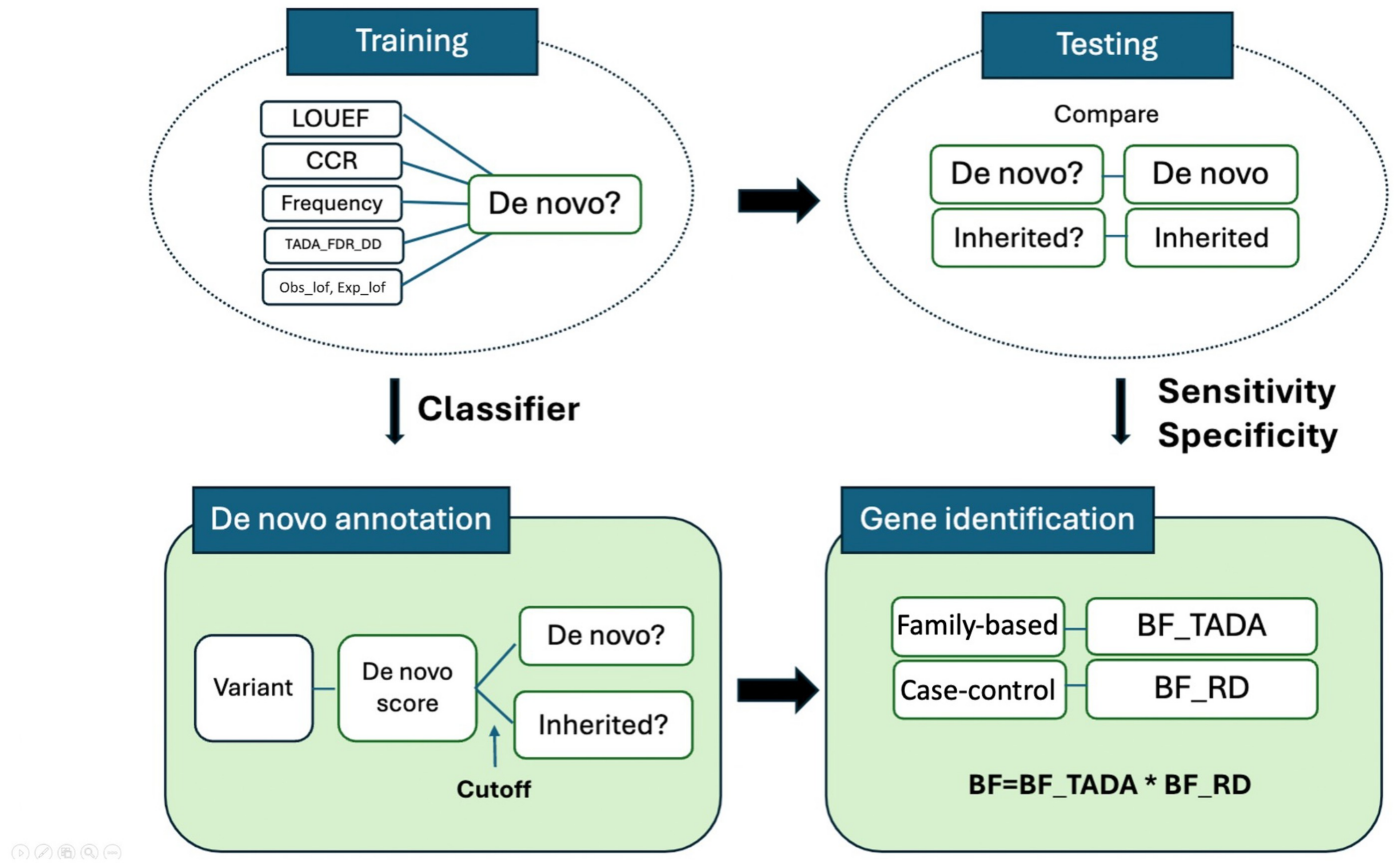
Overview of workflow and the datasets. (Top) Summary of dataset used in the data application. (Bottom) Flowchart of the method.

Next, we develop an approach to incorporate this information into a gene-based association framework and thereby garner greater power to identify relevant genes. We build on the TADA model (Transmitted and De novo Association) (He et al. 2013), which combines different types of data, including those of families with affected probands, probands without parental information and controls. Although TADA provides a comprehensive analytical framework to accumulate the signals from each kind of data and over types of variants (e.g. PTV, missense), it provides only a modest signal for association from case versus control data. If we can identify the *de novo* variants in cases, we conjecture that this will enhance the TADA signal and the power for association. In this manuscript, we propose a new integrated model that evaluates variation in case and control samples, attempts to sort *de novo* mutations from inherited variation, and incorporates this information into a refined TADA model (Fig. 1). We exemplify the method using published ASD data. Importantly, the approach we develop can be used to associate rare variants with any developmental phenotype, such as schizophrenia, obsessive-compulsive disorder, and congenital heart disease.

## Materials and methods

### Overview of workflow and the datasets

Our procedure comprises two separate pipelines (Fig. 1). The first builds *ClassDn*, a classifier to predict the inheritance class of variants (*de novo* or inherited) using offspring level variants with known inheritance class. *ClassDn* is then applied to test data to assess the classifier’s performance, which serves as a component of the input parameters for the data application. In the second pipeline, *ClassDn’*s predictions are applied to variants without parental information to obtain their likelihood of being *de novo*, called de novo scores. When applied to the full case sample, these scores are converted to gene-level risk scores by a new *Random Draw (RD)* model, designed to be robust to uncertainty in the predicted inheritance class and to control false discoveries at the gene-level. Combining this information with other information assimilated in the existing TADA framework (Fu et al. 2022) produces a more powerful gene-based association test.

Data from previously published exome sequencing data from ASD and control subjects are used for our analysis. These data, described in Fu et al. (2022), are derived from two sources: the Simons Powering Autism Research (SPARK) initiative and the Autism Sequencing Consortium (ASC) samples. In this study, we focus only on putative protein-truncating variants or PTV, because they are best understood in terms of their impact on gene function. Following Fu et al. (2022), we restrict our analysis to ultra-rare variants (<.1% population frequency). Both ASC and SPARK samples consist of family-based data, where the inheritance class of offspring variants is known, and case-control data, where inheritance is unknown.

To summarize, in the first pipeline, an unbalanced classification algorithm *ClassDn* is trained with SPARK family-based data (**training**) and then is tested on ASC family-based data to learn performance parameters (**testing**). In the second pipeline, *ClassDn* is applied to ASC case-control data to learn *inferred de novo* status of variants (**de novo annotation**), and *Random Draw model* is applied to them to obtain BF_RD for each gene. Multiplying BF_RD by BF_TADA (from ASC family-based data) yields the association score of genes (**gene identification**). For further details, see Supplementary Material A, available as supplementary data at *Bioinformatics* online.

### Classification algorithm for inheritance class

Regarding genetic association with ASD, the major signal arises from *de novo* PTVs; by comparison, the information emerging from case-control and inherited PTVs is relatively minor (Fu et al. 2022). Moreover, the signal derived from case-control samples is far greater than that from inherited variation assessed from family-based samples (Satterstrom et al. 2020), indicating that the case-control sample likely includes a non-negligible number of *de novo* and very recent mutations, which are typically more damaging. We conjecture that the distinct nature of *de novo* and inherited variants will allow us to separate them using only offspring genetic information, including both variant and gene-level variables. We use family-based data to build the *ClassDn* classifier to identify *inheritance class*.

**Figure 2:**
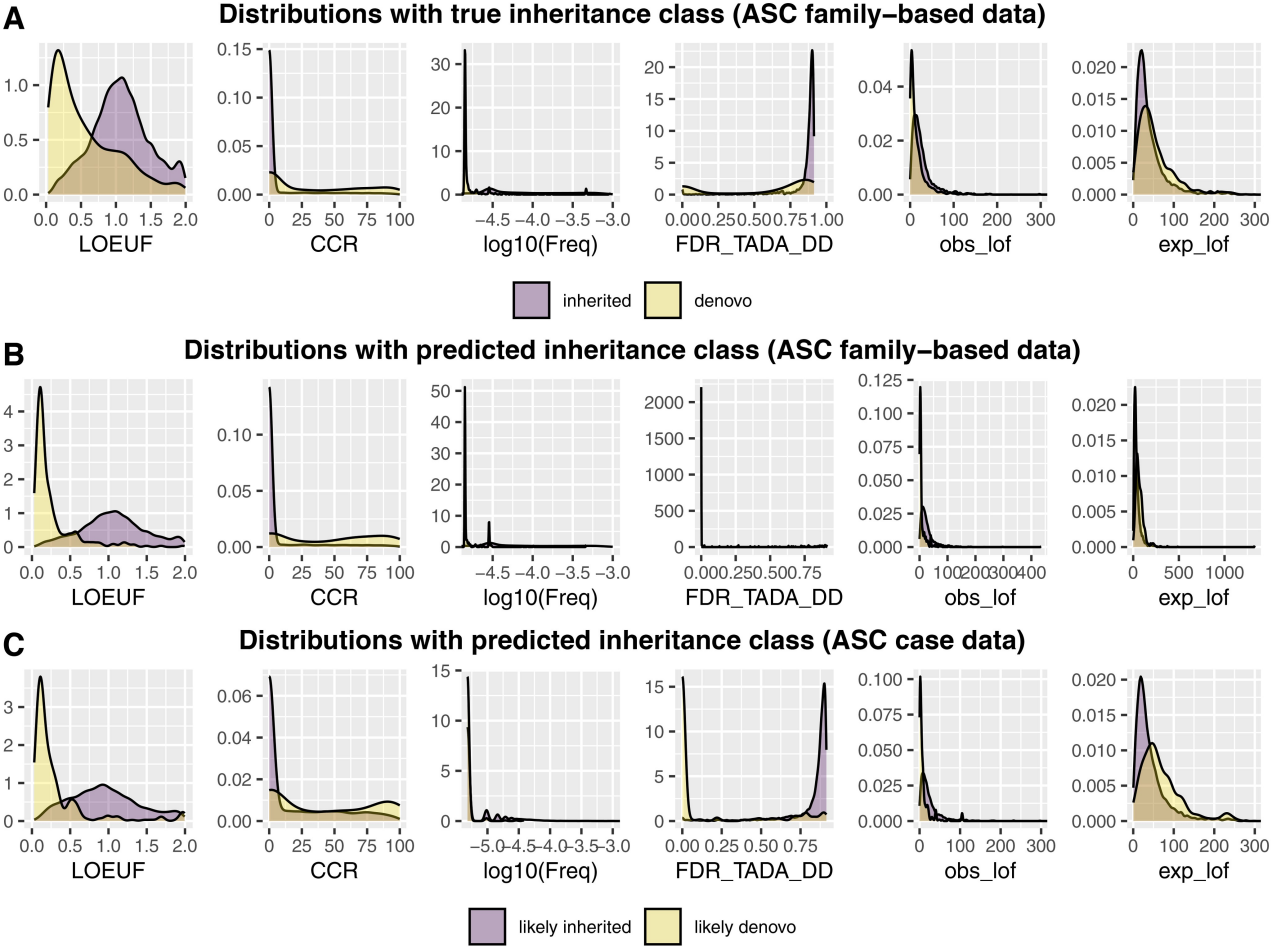
Six variables used in the analysis help distinguish *de novo* and inherited variants. A. Density plots of covariates for *de novo* and inherited variants in proband data from the ASC family-based data. B. Density plots of covariates for likely *de novo* and likely inherited variants in proband data from ASC family-based data. C. Density plots of covariates for likely *de novo* and likely inherited type of variants in ASC case data. RUSboost with a threshold 0.7 was utilized to generate the Figure 2B and 2C.

We incorporate six variables to infer the inheritance classes of variants: Allele Frequency (AF), LOEUF (Karczewski et al. 2020), CCR (Havrilla et al. 2019), FDR_TADA_DD, obs_lof and exp_lof. AF refers to the proportion of a particular allele (variant) in a population, which is obtained from the gnomAD library. Gene (LOEUF) and variant (CCR) scores are measures of evolutionary constraint against variation in the gene or region therein, due to its impact on reproductive success. Consequently, PTVs falling in these regions/genes are more likely to be *de novo* than inherited. A variant-level constraint score, the constrained coding region (CCR), measures depletion of protein-changing variants in specific coding regions; it ranges from 0 to 100. CCR scores are matched to variant locations using starting position of the CCR. The continuous gene-level score LOEUF is based on the observed and expected numbers of loss-of-function variants (obs_lof and exp_lof), which individually contribute to distinguishing *de novo* variants in classifiers. While LOEUF summarizes relative constraint, obs_lof and exp_lof separately provide additional information, capturing both relative constraint and absolute mutation burden. Incorporating these two components alongside LOEUF therefore allows the classifier to leverage complementary aspects of the underlying signal and leads to improved performance. Because ASD is often one of a constellation of symptoms of neurodevelopmental delay (NDD) or developmental delay (DD/NDD), genes affecting ASD and those affecting DD/NDD largely overlap (Doshi-Velez et al. 2014, Sanders et al. 2019). Therefore, we use the known risk score of gene associated to one of theses conditions FDR_TADA_DD (Fu et al. 2022). All six covariates effectively distinguish the inheritance class of variants (Fig. 2A). Compared to inherited variants, *de novo* variants exhibit a lower allele frequency, a lower LOEUF score, a higher CCR and a lower FDR score related to DD. Such distinctions confirm that a classifier based on these variables will generate meaningful separation of variants in terms of different inheritance classes.

Natural competitors to LOEUF are estimates of PTV selection coefficients (Weghorn et al. 2019, Agarwal et al. 2023). To evaluate whether incorporating an explicit selection-based measure improves our framework, we conducted an additional analysis in which LOEUF and CCR were replaced by the selection coefficient–based metric *hs*, which was estimated by Agarwal et al. (2023) for each of 17,318 autosomal genes. We use their reported maximum a posteriori (MAP) estimate. We matched each gene in our data to its corresponding transcript’s hs value; compared model performance using precision–recall curves under a range of tuning parameters; and found that models incorporating LOEUF and CCR consistently achieved higher training PR-AUC than models using the *hs* measure.

**Figure 3:**
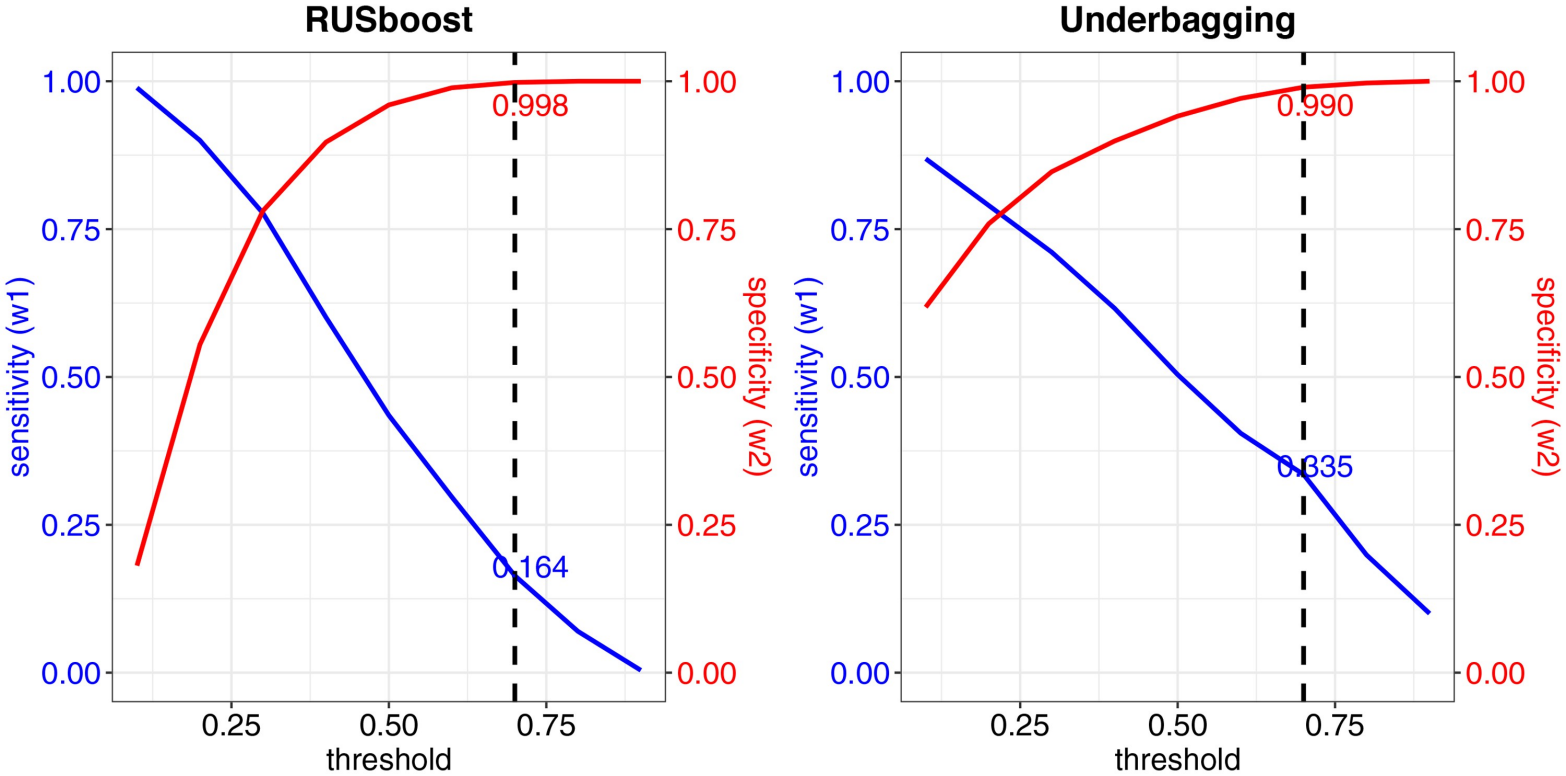
Sensitivity and Specificity of classifiers for a range of thresholds: RUSboost (left) and Underbagging (right). Results at the threshold of 0.7 are indicated by the dashed line.

Notably, the number of inherited variants greatly exceeds that of *de novo* variants. In such imbalanced data, a naive classifier neglects performance for the minority class data. Therefore, for *ClassDn*, we implemented algorithms specifically designed to handle imbalanced data, such as RUSBoost (Seiffert et al. 2010) and Underbagging (Barandela et al. 2003), both of which balance class prediction through resampling techniques. The algorithms output the likelihood of being *de novo* for each variant, termed de novo scores. Combining this with a specific choice of threshold, we classify a variant as “likely de novo” if its score exceeds the threshold and “likely inherited” otherwise. The overall performance of *ClassDn* depends on the choice of threshold and is summarized by two parameters: sensitivity (the probability that *de novo* variants are classified as *de novo*; $w_{1}$) and specificity (the probability that inherited variants are classified as inherited; $w_{2}$). A natural trade-off exists between sensitivity and specificity: using a higher threshold decreases sensitivity and increases specificity. For our data, good performance was achieved Fig. 3 with the following parameters: for RUSboost, with a choice of threshold 0.7, the sensitivity is 0.164 and the specificity is 0.998; for Underbagging, sensitivity is 0.335 and specificity is 0.990. The separation between two predicted classes is notable with six covariates (Fig. 2B, RUSBoost, with threshold 0.7, was used to generate this figure). This conservative algorithm results in slightly more extreme separation compared to the true inheritance class for some covariates.

### Identification of risk genes

The Transmission and De Novo Association (TADA) model is a Bayesian statistical framework to identify associated genes using variants of various types and inheritance classes (He et al. 2013, Fu et al. 2022), with each type and class yielding a Bayes Factor for association. Multiplication of independent BFs per gene integrates this information for a final association score. Genes with large scores are associated. Here we advance the TADA model by using *ClassDn* to interpret PTV variants found in individuals with ASD, but for whom parental data are missing (case-only data), and compute a BF for these data using the *Random Draw* model. This *Random Draw* BF replaces the corresponding case-control component of the original model.

Evaluating the results from Fu et al. (2022), we noted that genes with large *de novo* BFs tend to have fewer inherited variants and vice versa (see Fig. 2 available as supplementary data at *Bioinformatics* online). We induce this negative correlation in the *Random Draw* model by assuming that each variant is a random draw from a mixed pool of *de novo* and inherited variants of fixed sample size, and estimate the likelihood of the observed composition of *de novo* and inherited variants. Furthermore, each gene has a baseline probability of mutation (Fu et al. 2022), which is multiplied by γ>1 when the gene confers risk. Once we estimate a prior distribution for the probability of drawing a *de novo* variant under each risk and non-risk gene scenario, as described below, we can compute the likelihood for the *de novo* and inherited variants and then contrast the two scenarios using a Bayes factor. A remaining challenge for implementation is that the predicted inheritance class from *ClassDn* is only a proxy for the real inheritance status of variants. To incorporate such uncertainty, classification accuracy parameters are utilized.

Define *I* to be an indicator of *de novo* status of a given variant (1 for *de novo*  and 0 for inherited) and *X* to be its predicted inheritance class (1 if the ClassDn score is greater than the threshold, and 0 otherwise). For an indicator *D* of the risk status of the gene (1 for risk genes and 0 for non-risk genes), and a mutation rate μ, define $p_{1}^{\mu }=P(I=1|D=1,\mu )$ and $p_{0}^{\mu }=P(I=1|D=0,\mu )$ to be probability that the variant is *de novo* under each scenario ($p_{1}^{\mu }>p_{0}^{\mu }$). Suppose we observe counts of $\left(x_{d},x_{h}\right)$ variants for a given gene, where $x_{d}$ represents the number of likely de novo variants and $x_{h}$ represents the number of likely inherited variants, classified as greater than and less than the threshold, respectively. The likelihood of such data under each scenario are identified from the binomial distributions, as


 P(xh,xd|D=1) =(xh+xdxd)P(X=1|D=1)xd(1−P(X=1|D=1))xh P(xh,xd|D=0) =(xh+xdxd)P(X=1|D=0)xd(1−P(X=1|D=0))xh.

To account for uncertainty in classification, we denote the accuracy parameters as $w_{1}=P(X=1|I=1)$, the sensitivity, and $w_{2}=P(X=0|I=0)$, the specificity. For given values of $p_{0}=p_{0}^{\mu }$, $p_{1}=p_{1}^{\mu }$, the binomial parameters can be expressed as $P(X=1|D=1)=w_{1}p_{1}+(1-w_{2})(1-p_{1})$, and $P(X=1|D=0)=w_{1}p_{0}+(1-w_{2})(1-p_{0})$. The evidence for D=1 versus D=0 can be calculated as a Bayes factor. For details, including how to estimate gene specific mutation rates, see the Supplementary Material A, available as supplementary data at *Bioinformatics* online.

## Results

### Validation of the random draw model

The original TADA model model compares the counts of unlabeled variants in cases to controls to derive a case-control score (TADA${\;}_{CC}$). The *Random Draw* model (TADA${\;}_{RD}$) provides a new way to estimate risk scores from variants without parental information (case-only data), whereas it uses the same TADA framework for *de novo* variants. Even though the Random Draw model is applied only to case-control data in real applications, as a check for the validity of the new model, we compare the risk scores obtained from the original TADA model and the Random Draw model using only the family-based dataset (with $w_{1}=w_{2}=1$ for the Random Draw model). Despite the differences in formulation, the resulting risk scores show high consistency (Fig. S3, available as supplementary data at *Bioinformatics* online).

To verify that the Random Draw model enhances gene discovery while controlling false discoveries, we conduct simulations in two settings, no signal and expected signal. To evaluate data without signal, we first generate 100 data sets with attributes similar to the unaffected siblings in the ASC data (see Supplementary Material B, available as supplementary data at *Bioinformatics* online). For each null dataset, we apply our *ClassDn* method to obtain *de novo* scores for each variant, identify the number of likely *de novo* and inherited variants for each gene with a choice of threshold *c*, and then apply the Random Draw model. Per-gene Bayes factors (BFs) from each dataset are multiplied by per-gene BFs from the real ASC family-based results, which have signal. (This avoids calculating False Discovery Rates without signal.) Final BFs were jointly transformed to FDR q-values following the approach described in Li et al. (2010) (Section 2.3). Two cutoffs are used for gene selection; first, we convert q-values to *P*-values and select genes with *P*-values less than .05 divided by the total number of genes considered (Bonferroni procedure); second, we use FDR<0.05. For evaluation, the number of false selections (Per-family error rate: PFER) averaged over the datasets are reported. Because the simulated case data contain no disease signal, a good benchmark for assessing type I error is the set of results from the analysis of the ASC family data only (Fu et al. 2022). We define false discoveries as genes that are not in the set of 185 genes with FDR<0.05. Comparing results with and without the null case data allows us to assess whether type I error is inflated; of course, this assessment is somewhat conservative because some genes with FDR>0.05 have relatively large BF. Nonetheless, the benchmark PFER is 0 when using the Bonferroni cutoff and 8 when using a FDR cutoff of 0.05, while the Random Draw model with inferred *de novo* variants does not measurably inflate false discoveries for strict thresholds of c=0.7 or c=0.9 (Table 1).

**Table 1:** A random draw approach enhances power while maintaining reasonable control of false discoveries in simulation datasets.

	Null (PFER)				Power		
	RUSboost		Underbagging		$w_{1}$	$w_{2}$	Disc
c	Bonf	qval	Bonf	qval			
0.3	1.1	11	1.2	10.6	0.829	0.624	66.2
0.5	1	10.5	1.1	10.2	0.624	0.829	72.1
0.7	0.1	8.2	0.1	8.5	0.376	0.943	72.4
0.9	0	8	0	8	0.171	0.987	67.0

When only family-based data is considered, PFER is 0 with a bonferroni cutoff and 8 with the FDR cutoff of 0.05. The number of discovery is 58 when only family-based data is used, and 64 when case-control data is incorporated with TADA${\;}_{CC}$ model.

In the power simulation, we use the list of genes and their corresponding number of variants from the ASC case dataset and simulate the inheritance class and de novo score of each variant; in reality, this is hidden information in these data. Specifically, we assume that the fraction of *de novo* variants is 0.603 for genes with q-value <0.05 in the most informative data analysis published (Fu et al. 2022) and 0.026 otherwise. These values represent the proportion of *de novo* variants within the ASC family-based data for the risk gene and non-risk genes, respectively. Then the final numbers of *de novo* variants are computed as the product of these *de novo* fractions and the number of case variants of each gene, rounded to the nearest integer, and lower bounded by zero. The remaining variants are set as inherited variants. Based on the simulated inheritance class label for each variant, we assign a random de novo score generated from a Gaussian distribution, with a mean of 0.6 and a variance of 0.1 for *de novo* variants and a mean of 0.2 and a variance of 0.1 for inherited variants. In our analysis, we assume that the inheritance status of variants is unknown, but their de novo scores obtained from *ClassDn* are observable. For convenience, we further assume that the distribution of de novo scores is known in both *de novo* and inherited scenarios. This implies that once a threshold *c* is selected, the sensitivity and specificity parameters $w_{1}$ and $w_{2}$ can be computed from these distributions. In real-world applications, these parameters would need to be estimated using test data labeled with the true *de novo* status.

Upon generating the simulated data, we select a threshold level between 0.3 and 0.9 and apply it to the de novo scores: variants with scores exceeding the threshold are classified as likely de novo, while those below are classified as likely inherited. Given the sensitivity ($w_{1}$) and specificity ($w_{2}$) corresponding to the selected threshold, and the number of likely de novo and inherited variants per gene, Bayes factors are computed using the Random Draw model. The final BF is obtained by multiplying these BFs with those from the ASC family-based dataset, substituting any BF less than 1 with 1 to avoid down-weighting. We then convert the resulting BFs into FDR q-values and identify genes with q-values below 0.05. This procedure is repeated 100 times for each threshold level, and the average number of discoveries is reported. As in the null simulation, we use the result from a published dataset (Fu et al. 2022) as a proxy for the true risk gene status.

When the TADA model is applied to only ASC family-based data, the number of discoveries is 58. When case–control data are additionally incorporated through the original TADA framework, using the real ASC control data described in Fig. 1, TADA${\;}_{CC}$ identifies 64 genes. In comparison, TADA${\;}_{RD}$ provides a larger number of gene discoveries (Table 1), while a threshold of 0.7 yields the best results in terms of achieving strong power while controlling for errors.

The choice of threshold c = 0.7 plays an important role in interpreting TADA${\;}_{RD}$ results. This parameter was chosen in light of both the the sensitivity-specificity and power versus error control trade-offs. At this threshold the specificity has essentially achieved its maximum, while the sensitivity is clearly declining (Fig. 3). However, when paired with TADA${\;}_{RD}$, this conservative definition of “likely de novo” provides the best balance of performance (Table 1). In other applications, such as identifying the probable causal variants for a family, this choice may be overly conservative.

### Analysis of ASC case data with the random draw model

To analyze the case-only data, we set a threshold of 0.7 to de novo scores, which separates the variants into likely de novo and likely inherited sets using either of two types of *ClassDn* classifiers, RUSBoost and UnderBagging. Of the 13,486 PTV variants for case-only dataset, 2.2% (RUSBoost) and 3.7% (UnderBagging) of variants without parental information are classified as *de novo*. The *Random Draw* model then evaluates these pseudo-labeled data and generates the second BF using the sensitivity and specificity parameters learned from the ASC family-based dataset (Fig. 3). After ensuring that each BF exceeds or is set to a minimum of 1, the two BFs are multiplied. For the final discoveries, the BFs are converted to FDR values (q-values) following the approach described in Li et al. (2010) (Section 2.3). This procedure requires an estimated proportion of risk genes, for which a value of 0.06 was used. The final discovery is confirmed by selecting genes with a q-value less than 0.05. This entire procedure is referred to as TADA${\;}_{RD}$.

**Figure 4:**
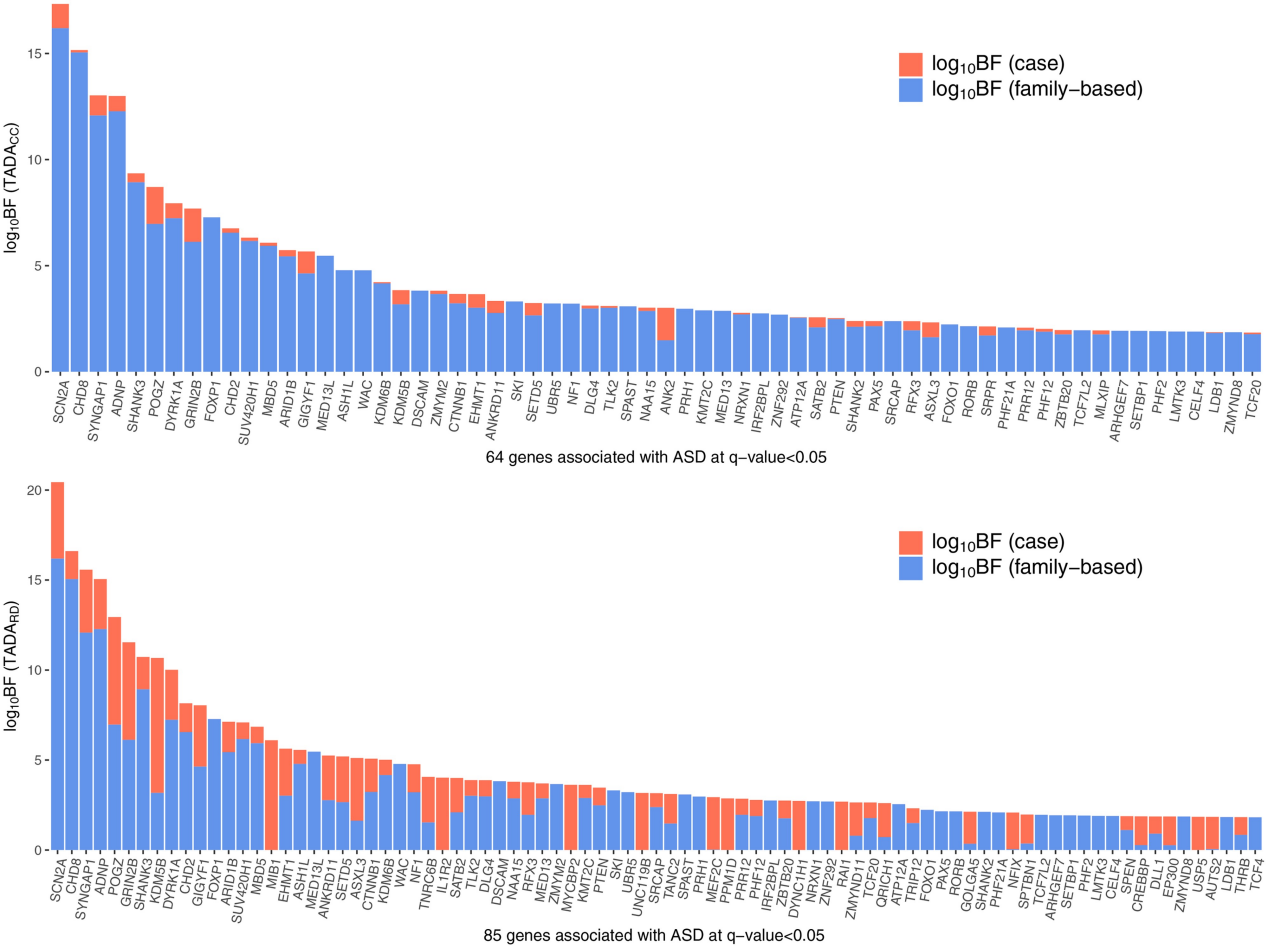
Integrating case data using the Random Draw model improves the association power among candidate genes. (Top) The evidence of ASD association contributed by each dataset for the 64 genes selected using the TADA${\;}_{CC}$ method with FDR ≤ 0.05. (Bottom) The evidence of ASD association contributed by each dataset for the 85 genes selected using the TADA${\;}_{RD}$ method with FDR ≤ 0.05. The result with RUSboost is shown. Most of the additional discoveries are validated by ongoing ASD cohort studies with larger datasets (unpublished data).

The results highlight that *ClassDn* effectively separates different inheritance classes of variants in terms of the six covariates used in the classifier (Fig. 2C). Likely de novo variants tend to have low LOEUF scores, high CCR, low frequency, low FDR_TADA_DD, low obs_lof, and high exp_lof, which is consistent with the properties of *de novo* variants observed in family-based data. Next, TADA${\;}_{RD}$ significantly enhances the discovery of gene associated with ASD, identifying a total of 85 risk genes for RUSboost and 103 for Underbagging, while the traditional TADA framework identifies 64. Because the final Bayes Factor is the product of two BFs, the contribution of each dataset to TADA${\;}_{RD}$ and TADA${\;}_{CC}$ is easily identified (Fig. 4; the result with RUSboost is shown). For TADA${\;}_{CC}$, the contribution of case-control data is typically modest. For TADA${\;}_{RD}$, however, the contribution is greatly increased, with case-only data contributing substantial signal for top-ranked genes such as *POGZ* and *GRIN2B*, which are considered high-confidence ASD genes (Fu et al. 2022).

Among the genes not selected by TADA${\;}_{CC}$, 23 are selected through both versions of *ClassDn* (Table S3, Supplementary Material C, available as supplementary data at *Bioinformatics* online). These selections are better understood by the number of likely de novo variants identified by *ClassDn*. Naturally, genes selected only by TADA${\;}_{RD}$ include at least one likely de novo variant in the case-only data. Some of these genes, such as *MEF2C* and *TNRC6B*, already exhibit moderate signal from TADA${\;}_{CC}$. However, for many other genes, case-only data serve as the primary source of the association signal: their q values are not even close to 0.05 in TADA${\;}_{CC}$, but are below 0.05 in TADA${\;}_{RD}$. This shows the critical boost in power of the new TADA model from inferred *de novo* variants and its capacity to elevate case-only data to one of the main sources of information for association.

## Conclusion and discussion

Detection of *de novo* LoF mutations can be a powerful means of discovering novel risk genes for various developmental disorders, including ASD (Fu et al. 2022) and congenital heart disease (Jin et al. 2017, Watkins et al. 2019). Yet, *de novo* events are relatively rare, roughly one per exome. Thus, if we can increase the number of *de novo* mutations identified in probands, especially for probands without complementary parental information, we should be able to increase the power to identify risk genes. To address this challenge, we propose a novel framework that integrates both family-based and case-control data using a classification algorithm to probabilistically infer inheritance status of variation found in subjects who do not have complementary parental data. Then, by integrating a classifier trained on family-based data with a principled gene-level association model (*Random Draw* model), we address the key challenge of missing inheritance labels in case-control or case-only datasets. Besides discovering additional risk genes, there is another key benefit of these methods. Using our classifier, likely *de novo* variants can be identified, which can be critical for clinical genetic evaluation when complete parental genotypes are unavailable.

Simulation studies demonstrate that this novel approach maintains control over false discoveries and can substantially enhance statistical power. Furthermore, when applied to exome sequencing data from ASD families and ASD case-control studies, the proposed method (TADA${\;}_{RD}$) substantially increases the number of risk gene discovered compared to the conventional TADA model. This increased power emerges by using case data more effectively, specifically identifying likely *de novo* variation and interpreting the resulting *de novo* score using TADA${\;}_{RD}$.

There are alternatives to TADA and RD, such as SKAT (Wu et al. 2011). SKAT, however, requires individual-level genotype data, which are often unavailable. For instance, our data were obtained from multiple sources, some of which do not permit sharing of individual-level genotype information. To overcome this challenge, we generated individual-level data by simulation. From the gene set used in the case–control analysis, we randomly selected risk genes from the larger set. Because SKAT requires sufficient variant counts, we restricted risk genes to be those with more than eight observed variants (combining cases and controls). *De novo* fractions for risk and non-risk genes were sampled from gene-specific Beta distributions, and variants were subsequently labeled as *de novo* or inherited accordingly. For a given gene, we generated individual-level genotypes under the assumption that approximately 80% of variants were singletons, consistent with our data, and allocated them randomly across loci and individuals. Variant effect sizes were generated from a normal distribution with mean zero and standard deviation equal to the specified effect size, with positive effects (0.3 or 0.5 on the log-odds scale) assigned to risk genes and zero otherwise. Individual phenotypes were generated under a logistic regression model, with the intercept chosen to yield approximately balanced case–control sample sizes. In this realistic setting, SKAT exhibits little power (data not shown). The limited power occurs because the rare-variant signal in ASD is driven by a small number of extremely rare, largely *de novo* variants. SKAT’s genetic similarity kernel, however, relies on more abundant inherited variants of modest (or larger) effect to power it.

A key parameter of the random draw method for risk gene discovery is the proportion of variants within a gene that are *de novo*. Our likelihood-based framework bins genes into those that are versus are not associated with ASD, using prior association results, and then estimates group-specific distributions of de novo fractions probabilistically from a model fitting gene-level covariates. As shown here, such a model can support association inference even for genes without observed de novo events. However, because our estimates are informed only by genes with prior evidence of association, it is likely a biased estimator, and this is a limitation of the current framework.

In theory, evolutionary selection against PTV variation (Weghorn et al. 2019, Agarwal et al. 2023), as captured in s_het, are natural replacements for biased measures such as LOEUF. These quantities provide an explicit evolutionary interpretation and, in principle, represent a natural baseline for modeling *de novo* enrichment. In practice, however, estimates of selection coefficients require imprecise modeling assumptions and are subject to uncertainty arising from limited sample sizes, mutation-rate calibration, and transcript annotation. Empirical constraint metrics such as LOEUF and CCR, while heuristic and not direct estimators of selection, summarize population-level depletion patterns that appear to capture similar information relevant for prediction. In our analyses, models incorporating LOEUF and CCR consistently achieved stronger discriminative performance than those using selection coefficient–based measures across a range of resampling and tuning settings, suggesting that empirical constraint scores, while biased, offer greater stability for predictive modeling in finite samples. Nevertheless, selection-based parameters remain conceptually central to understanding evolutionary processes, and continued improvements in their estimation—especially at the transcript and variant-class level—could enable more generalizable and mechanistically grounded estimates for future gene-based association research.

Of the 23 additional genes identified by both versions of *ClassDn*, all except four are found in SFARI Gene (2025 Q4), an authoritative resource for genes associated with ASD (Abrahams et al. 2013), and with scores implicating strong evidence for association. The exceptions are *GOLGA5*, *THRB*, *UNC119B*, and *USP5*. *GOLGA5* and *THRB* have 3 and 2 de novo PTVs in ASD probands (Fu et al. unpublished data), respectively, providing corroborating evidence for association. The deubiquitinase *USP5* plays roles in regulation of calcium channels (Antunes et al. 2025), and calcium ion genes have been linked to ASD (Fu et al. 2022). Insofar as we are aware, however, *UNC119B* has no supporting evidence beyond the case data, making it a plausible false positive. Curiously, another gene might also represent a false positive, although for a different reason. *MIB1* is in SFARI Gene that has also been associated with developmental disabilities (Fu et al. 2022). However, recent studies have shown that PTVs in *MIB1* are under positive selection in spermatogonia (Neville et al. 2025, Seplyarskiy et al. 2025). This observation calls into question the estimated mutation rate for *MIB1*, which is used to compute the expected number of mutations for TADA_RD and other association models.

Two genes become insignificant when using TADA${\;}_{RD}$: *MLXIP* and *SRPRA*. These could be false negatives. An additional 18 genes were identified by only Underbagging version of *ClassDn*. A large fraction of these 18 have support for association (16 out of 18, see Supplementary Material C available as supplementary data at *Bioinformatics* online). The difference between RUSBoost and Underbagging primarily reflects the more conservative behavior of RUSBoost under the same nominal threshold, rather than a substantive disagreement between the methods. The ranking of inferred de novo scores is highly consistent across the two classifiers, and the classifier-specific genes tend to lie near the significance boundary. Nonetheless, given these results, a conservative approach would be to use multiple classifiers to identify likely *de novo* variants.

Although this work focuses on protein-truncating variants, the framework is broadly applicable and can be extended to other variant classes and phenotypes. Our approach highlights the value of incorporating probabilistic annotations of *de novo* status into association models, and offers a scalable solution for maximizing information from incomplete but abundant sequencing datasets.

## Supplementary Material

btag177_Supplementary_Data

## Data Availability

The implementation code and publicly available data are available at Github (https://github.com/HaeunM/TADA-RD) and Zenodo (DOI: https://doi.org/10.5281/zenodo.18531769). Due to GitHub file-size constraints, a small number of large auxiliary files, which are not required to execute the main analysis pipeline, are provided in Supplementary Material D for completeness.
