# Supplementary material for: A framework to infer de novo exonic variants when parental genotypes are missing enhances association studies of autism
Source: Bioinformatics. 2026 May 4;42(5):btag177. doi: 10.1093/bioinformatics/btag177 (PMC13151883; doi:10.1093/bioinformatics/btag177)
Supplement: btag177_Supplementary_Data [file btag177_supplementary_data.zip › final_version_sup.pdf]

Supplementary Material A : Method Details

1.1 Data preprocessing

De novo data We obtained all de novo and inherited variant data from Fu et al. (2022) for ASC and SPARK, separately. All *de novo* variants incorporated in this study were used directly as provided. Additional postprocessing filters were applied to inherited variants so that the final dataset was enriched for true associations. Specifically, we added an additional within-family segregation filter: variants were retained only if they were transmitted exclusively to affected children or exclusively to unaffected siblings, but not to both. This approach is based on the assumption that ultra-rare variants transmitted to both affected and unaffected siblings are unlikely to meaningfully contribute to autism risk. To further improve signal quality, we filtered inherited variants to those with allele frequency *leq* 0.00005 in the non-neuro subset of gnomAD v2.1.1 and *leq* 0.0001 in their respective internal datasets. Inherited PTVs were only retained if classified as high-confidence (HC) by the LOFTEE plugin for VEP and allowed only the "SINGLE_EXON" flag among LOFTEE annotations. All variants were subsequently annotated with CCR scores (Havrilla et al., 2019), gene-level LOEUF metrics (Karczewski et al., 2020), expected and observed predicted loss-of-function variant counts (exp_lof and obs_lof, respectively), Developmental Delay FDR scores (Fu et al., 2022) and gnomad non-neuro AF, when available. Otherwise, variant frequencies were approximated within the appropriate dataset. Note that CCR coordinates were printed in bedfiles, which begin incrementing at 0. Therefore, we added 1 to all start and end coordinates to ensure compatibility with all VCF files.

Case-Control data The case–control data analyzed here were drawn from ASC cohorts originally sequenced and described in prior large-scale exome studies (De Rubeis et al., 2014; Lim et al., 2017; Satterstrom et al., 2020), and subsequently incorporated into the unified analysis reported by Fu et al. (2022). These data include samples from ASC sites in Japan, the population-based Swedish PAGES cohort (Population-Based Autism Genetics & Environment Study), and from the National Institute of Mental Health (NIMH) genomics repository. The Japanese cohort comprised 193 ASD cases and 298 unaffected controls (Lim et al., 2017), with diagnoses established using standardized assessments including the Autism Diagnostic Interview–Revised (ADI-R) and DSM-V criteria, and was supported by funding from the Japan Agency for Medical Research and Development (AMED; grants JP18dm0107087 and JP18dm0207005). The NIMH cohort included 450 ASD cases and 365 controls (Neale et al., 2012; Lim et al., 2013), with diagnostic ascertainment based on established instruments including the ADI, Autism Diagnostic Observation Schedule (ADOS), Mullen Scales of Early Learning, and Raven's Coloured Progressive Matrices. The Swedish PAGES cohort comprised 727 ASD cases and 3,586 controls and has been described previously (Gaugler et al., 2014) as an epidemiological ascertained community-based sample drawn from national registers, with ASD diagnoses based on ICD-10 criteria; this cohort was supported by funding from the Seaver Foundation and the National Institute of Mental Health (R56 MH097849; R01 MH097849). Across ASC cohorts, ASD diagnoses were established using comparable ascertainment and diagnostic protocols, as described in the original cohort publications (see Supplementary tables, Cohort Overview in Satterstrom et al. (2020) and Supplementary Table 1 in De Rubeis et al. (2014).

Variants were annotated with allele frequency data from gnomAD v2.1.1; when unavailable, allele frequencies were approximated using internal cohort-specific estimates. Consistent with the approach used for filtering and annotating de novo variation, case-control variants were also filtered using a non-neuro subset of gnomAD v2.1.1 allele frequency threshold of *leq* 0.001. As with the trio data, all variants were subsequently annotated with CCR scores (Havrilla et al., 2019), gene-level LOEUF metrics (Karczewski et al., 2020), expected and observed predicted loss-of-function variant counts (exp_lof and obs_lof), Developmental Delay FDR scores (Fu et al., 2022), and gnomAD non-neuro allele frequencies, when available.

1.2 Details of the classifier training procedure

We use rare variant information from the SPARK family-based dataset, which includes data from 7,008 families. Each variant in this dataset is labeled with its inheritance class and accompanied by six offspring-level covariates that help distinguish between these classes. The fraction of *de novo* variants is approximately 0.0063 times that of inherited variants. In such scenarios, a naive application of a classifier tends to assign most variants to the inherited group—the majority class. While this approach may achieve a reasonable overall accuracy, it is not effective for the actual purpose of classification. This highlights the need for algorithms specifically designed to handle imbalanced data. Such algorithms typically rely on ensembling results from multiple learners, each trained on a subset of samples that are balanced through either oversampling the minority class or undersampling the majority class.

After an initial screening of various ensemble algorithms, we selected two methods that use random undersampling of the majority class: RUSBoost (Seiffert et al., 2009) and Underbagging (Barandela et al., 2003). A key difference between these two algorithms is that RUSBoost iteratively selects training samples for each learner, assigning greater weight to samples that were misclassified by previous learners. In contrast, Underbagging builds learners in parallel, each trained on a randomly under-sampled, balanced subset of the data. These two approaches correspond to distinct algorithmic paradigms—boosting and bagging—which address class imbalance through fundamentally different mechanisms. In preliminary analyses, we observed that alternative methods within the same paradigm exhibited broadly similar performance, without substantive differences in the resulting conclusions. Therefore, selecting one representative method from each family allows us to capture the main methodological contrasts relevant to imbalance while avoiding redundant comparisons among closely related algorithms.

The outputs of these algorithms are scores ranging from 0 to 1, where a higher score indicates that a variant is more likely to belong to the de novo class. A specific threshold can then be applied to these scores to complete the classification procedure. For example, applying a threshold of 0.7 means that a variant is classified as de novo only if its score exceeds 0.7.

RUSBoost and Underbagging require several tuning parameters. The imbalance ratio parameter refers to the intended imbalance ratio for each learner, defined as the ratio of majority instances to minority instances after class rebalancing. The size parameter specifies the number

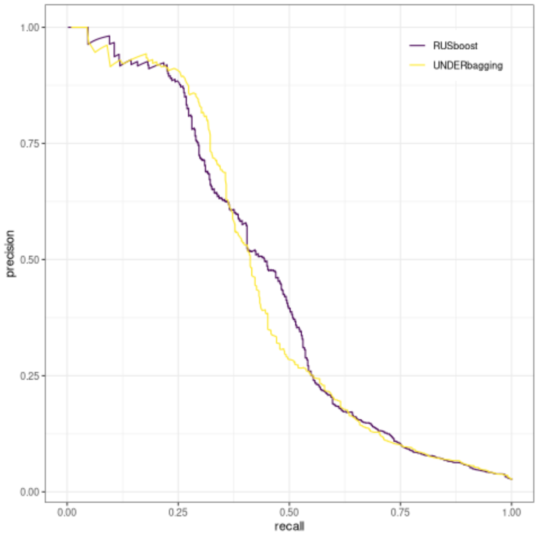

Figure S1 PR curve for parameters imbalance ratio=2, size=30, ntree=20 (fold 1)

Model	Imbalance ratio	Size	ntree	Mean.PR.AUC
rusboost	1	10	20	0.404
rusboost	1	30	50	0.413
rusboost	1.5	10	20	0.427
rusboost	1.5	20	50	0.437
rusboost	2	20	50	0.451
rusboost	2	30	20	0.454
rusboost	2	30	100	0.453
underbagging	1	10	20	0.411
underbagging	1	30	50	0.426
underbagging	1.5	10	20	0.434
underbagging	1.5	20	50	0.443
underbagging	2	20	50	0.454
underbagging	2	30	20	0.457
underbagging	2	30	100	0.459

Table S1 Mean PR AUC result for parameter selections.

of learners. Additionally, a choice of algorithm is required to construct the learners. We use the random forest algorithm, which includes a parameter for selecting the number of trees. To select these parameters, we divided the dataset into five equally sized folds. Four folds were used to train the model, and the remaining fold was used for validation. The performance metric was constructed based on two scores: precision and recall, defined as:

$$\text{Precision} = \frac{\text{True positives}}{\text{True positives} + \text{False positives}} \text{ and } \text{Recall} = \frac{\text{True positives}}{\text{True posi tives} + \text{False Negatives}},$$

where we define *de novo* class as positive. Tested parameters include ir=1, 1.5, 2 ; es=10, 20, 30; ntree=20, 50, 100 (where appropriate). Note that the LOEUF composition of variants in each bin was roughly identical by design. The final decision on the tuning parameters was based on the area under the Precision-Recall (PR) curve, which evaluates the performance of binary classification algorithms across different threshold levels (Figure S1). The final AUC value is averaged over the results from five test folds.

The results show that the mean area under the PR curve does not vary greatly across different parameter choices, illustrating that the classifier is robust to the selection of parameters (Table S1). Considering both performance and computational complexity, we selected an imbalance ratio of 2, ensemble size of 30, and number of trees per learner as 20 for both RUSBoost- and Underbagging-based classifiers. The PR curves for this set of parameters are also largely similar between RUSBoost and Underbagging, suggesting that the overall performance of the two algorithms is comparable (Figure S1). In the main analysis, when we tested the trained classifiers on the ASC family-based data, we found that RUSBoost identified fewer *de novo* variants than Underbagging at the same threshold. This difference does not indicate that Underbagging outperforms RUSBoost; rather, it reflects that the same threshold corresponds to a lower point on the recall axis for the RUSBoost-based classifier.

1.3 Details of the Random Draw Model

The random draw model first constructs the likelihood of having observed values of likely de novo and likely inherited variants under each risk gene and non-risk gene scenario. It then calculates a Bayes factor, defined as the ratio of this two likelihoods, as the evidence supporting the gene being a risk gene.

1.3.1 Background of the random draw model

The $TADA$ models is a well-established framework to model the number of *de novo* and inherited variants based on genetic parameters under both risk gene and non-risk gene scenarios. Both *de novo* and inherited variants contribute to the evidence supporting the gene being risky. A likelihood ratio, based on the observed number of variants, combined with estimated genetic parameters, provides a risk score for a specific gene. While both de novo and inherited variants contribute to gene-level evidence, results from Fu et al. (2022) show that gene risk is positively associated with the number of de novo variants and negatively associated with the number of inherited variants (Figure S2). Most risk genes therefore harbor relatively more de novo and fewer inherited variants.

The random draw approach captures this marginal relationship by modeling each variant as drawn from a mixture of de novo and inherited variants and evaluating the likelihood of the observed composition. Given a fixed total number of variants, a higher proportion of de novo variants thus implies stronger evidence that a gene is a risk gene.

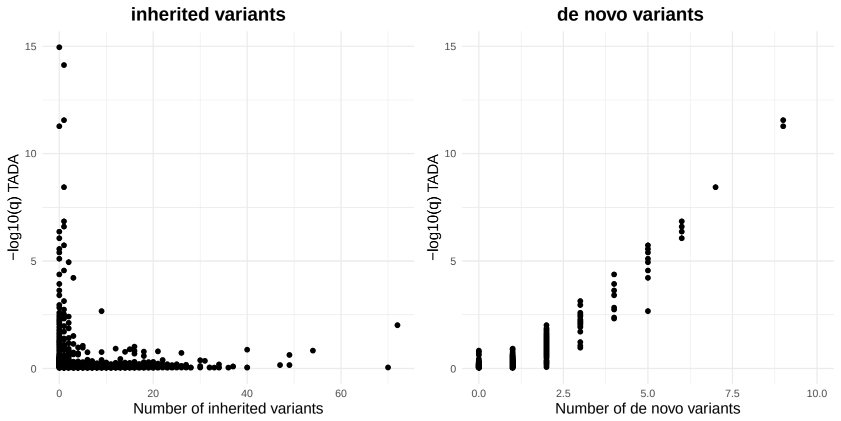

Figure S2 Marginal relationships between gene risk scores and the number of variants per gene: the number of inherited variants (left) and the number of de novo variants (right). Gene risk scores are represented as -log(q-value) computed in Fu et al. (2022).

With this approach, we can effectively deal with the uncertainty of the inheritance class involved in the case data, as explained in the subsection below. Additionally, even though this approach takes a different perspective to model the observed numbers of variants, it remains highly consistent with the original TADA model when there is no uncertainty regarding the inheritance class (see Figure S3).

1.3.2 Formulation

Let D_i denote the risk status of the gene i, where $D_i = 1$ if the ith gene is a risk gene and $D_i = 0$ otherwise. Let x_{d_i} and x_{h_i} denote the number of likely de novo and likely inherited variants, respectively, oberved in gene i. Also, let l_i^j denote the likely denovo status of the single variant j of gene i; $l_i^j = 1$ if it is likely de novo and $l_i^j = 0$ if it is likely inherited. Then the likelihood of observing x_{d_i}, x_{h_i} under risk and non-risk gene scenarios are formulated as follows:

$$P(x_{h_i}, x_{d_i}|D_i = 1) = \binom{x_{h_i} + x_{d_i}}{x_{d_i}} P(l_i^j = 1|D_i = 1)^{x_{d_i}} (1 - P(l_i^j = 1|D_i = 1))^{x_{h_i}} \tag{1}$$

$$P(x_{h_i}, x_{di}|D_i = 0) = \binom{x_{h_i} + x_{d_i}}{x_{d_i}} P(l_i^j = 1|D_i = 0)^{x_{d_i}} (1 - P(l_i^j = 1|D_i = 0))^{x_h}. \tag{2}$$

The quantities $P(l_i^j = 1|D_i = 1)$ and $P(l_i^j = 1|D_i = 0)$ denotes the probability that randomly selected variant is likely de novo for risk genes and non-risk genes, respectively. Let m_i^j be the indicator of variants being *de novo*, where $m_i^j = 1$ if *de novo* and $m_i^j = 0$ otherwise. Then we have

$$P(l_i^j = 1|D_i = 1) = P(l_i^j = 1|m_i^j = 1, D_i = 1)P(m_i^j = 1|D_i = 1) + P(l_i^j = 1|m_i^j = 0, D_i = 1)P(m_i^j = 0|D_i = 1),$$
$$P(l_i^j = 1|D_i = 0) = P(l_i^j = 1|m_i^j = 1, D_i = 0)P(m_i^j = 1|D_i = 0) + P(l_i^j = 1|m_i^j = 0, D_i = 0)P(m_i^j = 0|D_i = 0).$$

Here we assume that the probability that a *de novo* variant is observed as likely de novo and a inherited variant is observed as likely inherited is indifferent between risk and nonrisk genes, that is :

$$P(l_i^j = 1|m_i^j = 1) = P(l_i^j = 1|m_i^j = 1, D_i = 1) = P(l_i^j = 1|m_i^j = 1, D_i = 1)$$

$$P(l_i^j = 0|m_i^j = 0) = P(l_i^j = 0|m_i^j = 0, D_i = 1) = P(l_i^j = 0|m_i^j = 1, D_i = 0).$$

Then, we can compute the probability that some randomly drawn variant from gene i is likely denovo:

$$P(l_i^j = 1|D_i = 1) = P(l_i^j = 1|m_i^j = 1)P(m_i^j = 1|D_i = 1) + P(l_i^j = 1|m_i^j = 0)P(m_i^j = 0|D_i = 1),$$
$$P(l_i^j = 1|D_i = 0) = P(l_i^j = 1|m_i^j = 1)P(m_i^j = 1|D_i = 0) + P(l_i^j = 1|m_i^j = 0)P(m_i^j = 0|D_i = 0).$$

Two performance parameters $P(l_i^j = 1|m_i^j = 1) := w_1$ and $P(l_i^j = 0|m_i^j = 0) := w_2$ denotes the sensitivity and specificity of the classifier, respectively. They can be estimated empirically in a test sample (ASC family-based data in our application). Also, the quantities $p_i^1 = P(m_i^j = 1|D_i = 1)$ and $p_i^0 = P(m_i^j = 1|D_i = 0)$ are genetic parameters representing the probabilities that a variant drawn from gene i is a de novo variant, under the risk-gene and non-risk-gene scenarios, respectively. The parameter p_i^1 is expected to be higher than p_i^0. For given p_i^0, p_i^1, we

have

$$
\begin{aligned}
&P(l_i^j = 1|D_i = 1, p_i^1) \\
&= P(l_i^j = 1|m_i^j = 1)P(m_i^j = 1|D_i = 1, p_i^1) + P(l_i^j = 1|m_i^j = 0)P(m_i^j = 0|D_i = 1, p_i^1) \\
&= w_1 p_i^1 + (1 - w_2)(1 - p_i^1),
\end{aligned}
$$

and

$$
\begin{aligned}
&P(l_i^j = 1|D = 0, p_i^0) \\
&= P(l_i^j = 1|m_i^j = 1)P(m_i^j = 1|D_i = 0, p_i^0) + P(l_i^j = 1|m_i^j = 0)P(m_i^j = 0|D_i = 0, p_i^0) \\
&= w_1 p_i^0 + (1 - w_2)(1 - p_i^0),
\end{aligned}
$$

In our model, the parameters p_i^0 and p_i^1 follows some nondegenerate distributions under each risk and nonrisk scenarios: $p_i^0 \sim P_{0i}$ and $p_i^1 \sim P_{1i}$. To estimate these distributions, we specify the prior distributions $p_{0i} \sim Beta(\alpha_{0i}, \beta_{0i})$ and $p_{1i} \sim Beta(\alpha_{1i}, \beta_{1i})$ and estimate the gene-specific hyperparameters as follows. We group the genes based on their pre-risk status, which are determined by whether the q-values from the TADA model applied to only family-based data are above or below 0.05 (Fu et al., 2022). Then, for each pre-risk and pre-nonrisk gene sets, we fit a logistic regression model for the *de novo* ratio against log_{10}(mutation rate) for each gene, and use the predicted values to represent the mean values μ_{0i} or μ_{1i} of the distributions P_{0i} or P_{1i}. The variance of P_{0i} or P_{1i} are estimated through a jackknife approach, separately, under the assumption that the variance are identical among genes within risk or nonrisk sets. To calculate such variances, we exclude one gene at a time and compute a mean ratio as the total number of *de novo* variants divided by the total number of variants in each pre-risk/pre-nonrisk group. Let $\bar{x}_{1i}$ be the estimated mean of the *de novo* ratio calculated for genes in each group excluding the ith gene, and let $\mathcal{I}_{\text{risk}}$ and $\mathcal{I}_{\text{nonrisk}}$ are index sets for the pre-risk and pre-nonrisk genes . Then, the jackknife estimator for the variance is provided by

$$
\sigma_{0,jack}^2 = \frac{n-1}{n} \sum_{i \in \mathcal{I}_{\text{nonrisk}}} (\bar{x}_{1i} - \frac{1}{n} \sum_{i=1}^{n} \bar{x}_{1i})^2
$$

$$
\sigma_{1,jack}^2 = \frac{n-1}{n} \sum_{i \in \mathcal{I}_{\text{risk}}} (\bar{x}_{1i} - \frac{1}{n} \sum_{i=1}^{n} \bar{x}_{1i})^2.
$$

Then we convert gene-specific means and variance using formulas for beta distribution parameters;

$$
\alpha_{0i} = \mu_{0i} \left(\frac{\mu_{0i}(1 - \mu_{0i})}{\sigma_{0,jack}^2} - 1 \right), \beta_{0i} = (1 - \mu_{0i}) \left(\frac{\mu_{0i}(1 - \mu_{0i})}{\sigma_{0,jack}^2} - 1 \right)
$$

$$
\alpha_{1i} = \mu_{1i} \left(\frac{\mu_{1i}(1 - \mu_{1i})}{\sigma_{1,jack}^2} - 1 \right), \beta_{1i} = (1 - \mu_{1i}) \left(\frac{\mu_{1i}(1 - \mu_{1i})}{\sigma_{1,jack}^2} - 1 \right)
$$

Finally, the evidence of $D = 1$ against $D = 0$ can be calculated as a Bayes Factor, which accounts for uncertainty in the parameters (p_{0i}, p_{1i}) using gene-specific prior distributions P_{0i} and P_{1i}:

$$
BF_{i,RD} = \frac{P(x_h, x_d|D_i = 1)}{P(x_h, x_d|D_i = 0)} = \frac{\int P(x_{h_i}, x_{d_i}|D_i = 1, p_i^1)dP_{1i}}{\int P(x_{h_i}, x_{d_i}|D_i = 0, p_i^0)dP_{0i}}, \tag{3}
$$

where the likelihoods are integrated over the distribution of p_i^1 and p_i^0.

1.3.3 Robust check for the random draw model

To further validate the model, we compare the result from the random draw model and the original TADA model. In our main analysis, the random draw model was only applied to a case data, while the evidence from the family-based data is still collected through the family-based component of the $TADA_{CC}$ data. That is, in our main implementation, the only difference between $TADA_{RD}$ and $TADA_{CC}$ model is the treatment of the case-control data part. However, the random draw model can be still applied to a family-based data by simply setting $w_1 = w_2 = 1$. Since the TADA model has been validated in numerous previous studies for its validity and effectiveness, the comparison between the TADA model and the random draw model for the family-based data will serve as a tool to assess the robustness of our random draw model. A high correlation between two different approaches supports the validity of both methods (see Figure S3).

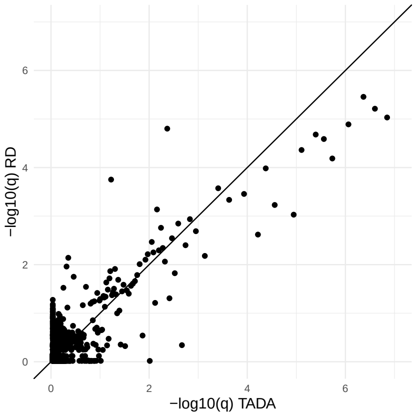

Figure S3 Comparison of the Random Draw and the TADA model in family-based data.

References

Barandela, R., Valdovinos, R. M., and Sánchez, J. S. (2003). New applications of ensembles of classifiers. *Pattern Analysis & Applications*, 6:245–256.

De Rubeis, S., He, X., Goldberg, A. P., Poultney, C. S., Samocha, K., Ercument Cicek, A., Kou, Y., Liu, L., Fromer, M., Walker, S., et al. (2014). Synaptic, transcriptional and chromatin genes disrupted in autism. *Nature*, 515(7526):209–215.

Fu, J. M., Satterstrom, F. K., Peng, M., Brand, H., Collins, R. L., Dong, S., Wamsley, B., Klei, L., Wang, L., Hao, S. P., et al. (2022). Rare coding variation provides insight into the genetic architecture and phenotypic context of autism. *Nature genetics*, 54(9):1320–1331.

Gaugler, T., Klei, L., Sanders, S. J., Bodea, C. A., Goldberg, A. P., Lee, A. B., Mahajan, M., Manaa, D., Pawitan, Y., Reichert, J., et al. (2014). Most genetic risk for autism resides with common variation. *Nature genetics*, 46(8):881–885.

Havrilla, J. M., Pedersen, B. S., Layer, R. M., and Quinlan, A. R. (2019). A map of constrained coding regions in the human genome. *Nature genetics*, 51(1):88–95.

Karczewski, K. J., Francioli, L. C., Tiao, G., Cummings, B. B., Alföldi, J., Wang, Q., Collins, R. L., Laricchia, K. M., Ganna, A., Birnbaum, D. P., et al. (2020). The mutational constraint spectrum quantified from variation in 141,456 humans. *Nature*, 581(7809):434–443.

Lim, E. T., Raychaudhuri, S., Sanders, S. J., Stevens, C., Sabo, A., MacArthur, D. G., Neale, B. M., Kirby, A., Ruderfer, D. M., Fromer, M., et al. (2013). Rare complete knockouts in humans: population distribution and significant role in autism spectrum disorders. *Neuron*, 77(2):235–242.

Lim, E. T., Uddin, M., De Rubeis, S., Chan, Y., Kamumbu, A. S., Zhang, X., D'Gama, A. M., Kim, S. N., Hill, R. S., Goldberg, A. P., et al. (2017). Rates, distribution and implications of postzygotic mosaic mutations in autism spectrum disorder. *Nature neuroscience*, 20(9):1217–1224.

Neale, B. M., Kou, Y., Liu, L., Ma'Ayan, A., Samocha, K. E., Sabo, A., Lin, C.-F., Stevens, C., Wang, L.-S., Makarov, V., et al. (2012). Patterns and rates of exonic de novo mutations in autism spectrum disorders. *Nature*, 485(7397):242–245.

Satterstrom, F. K., Kosmicki, J. A., Wang, J., Breen, M. S., De Rubeis, S., An, J.-Y., Peng, M., Collins, R., Grove, J., Klei, L., et al. (2020). Large-scale exome sequencing study implicates both developmental and functional changes in the neurobiology of autism. *Cell*, 180(3):568–584.

Seiffert, C., Khoshgoftaar, T. M., Van Hulse, J., and Napolitano, A. (2009). Rusboost: A hybrid approach to alleviating class imbalance. *IEEE transactions on systems, man, and cybernetics-part A: systems and humans*, 40(1):185–197.

Torrico, B., Shaw, A. D., Mosca, R., Vivó-Luque, N., Hervás, A., Fernàndez-Castillo, N., Aloy, P., Bayés, M., Fullerton, J. M., Cormand, B., et al. (2019). Truncating variant burden in high-functioning autism and pleiotropic effects of lrp1 across psychiatric phenotypes. *Journal of Psychiatry and Neuroscience*, 44(5):350–359.

Supplementary Material B : Simulation of Denovo and Inherited PTV in Siblings

This section describes the construction of a realistic pool of protein-truncating variants (PTVs) and the simulation of *de novo* and inherited PTVs in siblings, designed to mimic the distributions observed in the Autism Sequencing Consortium (ASC). The procedure integrates transcript annotations, population variation data, mutation rates, and conserved coding region (CCR) annotations, and produces variant-level datasets used in downstream analyses.

2.1 Construction of the PTV Variant Pool

A list of 80,950 transcripts representing 19,724 genes was obtained from the "LOUEF file" (Supplementary Dataset 11 in https://www.nature.com/articles/s41586-020-2308-7), downloaded 11/21/2023. After removing 1776 transcripts for which no LOEUF score was available, 79,174 (19,233 genes) remained. Subsequently, biomaRt (accessed on 05/12/2025) was used to determine: 1) whether the transcript was still supported; 2) the genomic boundaries of the transcript based on build38 (HG38); and 3) whether the transcript is the canonical transcript for the gene. The biomaRt-based filter reduced the number of transcripts to 71,876 (19,004 genes).

We then processed variant information from the "gnomAD file" (https://gnomad.broadinstitute.org/), downloaded 05/27/2025). The chromosome files were screened for PTV variants using the following criteria: 1) PASS field equals PASS; 2) IMPACT equals HIGH; 3) BIOTYPE equals protein coding; 4) Exonic equals T; 5) minor allele frequency based on non-neuro samples < 0.001; and 6) non-missing consequence. This screen of gnomAD data yielded 323,264 PTV variants in 17,376 transcripts representing 16,372 genes, with locations of variants from build38 (HG38). PTV-variants were classified as frameshift, stop gained, stop lost, start lost, splice acceptor, or splice donor. The number of overlapping transcripts, aligning gene and variant information, was 15,278. This set represents 14,557 unique genes and a total of 282,766 PTV variants. For 117 transcripts, the Ensembl gene id reported in the LOUEF file did not match the id reported by biomaRt. Removing these transcripts resulted in a set of 15,161 transcripts, 14,461 genes, and 281,049 PTV variants. Based on the biomaRt information, 10,146 of the transcripts were identified as canonical. An additional 1464 transcripts were identified as canonical by the LOEUF information. For each gene a single transcript was chosen to represent it by canonical status (TRUE over FALSE). Ties were broken by the length of coding sequence of the transcript (longer over shorter). This led to a list of 14,461 genes and transcripts and 273,042 variants.

Gene specific mutation rates for PTV based on ASC samples were obtained from the Fu paper Supplementary Table 5 (downloaded: 2022-09-28). Matches with our data could be made for 14,451 genes. For the remaining 10 genes, the mutation rates reported in the LOUEF data were used. Information on 8,188,409 conserved coding regions (CCR) was obtained from the file ccrs.autosomes.v2.20180420.bed.gz (https://www.nature.com/articles/s41588-018-0294-6, downloaded 02/25/2025). After removing genes without matching Ensembl gene id, 8,107,516 regions in 17,105 genes remained. Because the CCR locations were provided based on build37 (HG19), the liftOver script (downloaded: 2021-11-21) was used to perform a lift-over to build38 (HG38). Proper lift-over of genomic coordinates to build38 (HG38) could be obtained for 8,106,743 CCR regions.

Some final quality control of the data removed 473 variants that were not within the genomic boundaries of a canonical transcript, resulting in 272,929 PTVs variants representing 14,447 genes (transcripts). These will be the pool of PTVs for sampling in the simulations described below. CCRs were subsequently aligned with PTVs based on location. When a PTV mapped to multiple CCR regions, the maximum ccr_per was used. CCR values could be aligned with 244,734 PTV variants. The remaining 25,195 received a missing value.

2.2 Simulating Sibling Genotypes.

We next generated data consisting of rare *de novo* and inherited PTV variants for siblings of ASD subjects, with the goal of creating data similar to that observed from the ASC. In their sibling data, the ASC found 5374 PTV variants in 3768 genes, 5121 inherited, 189 d*de novo* and 4 were both inherited and *de novo* (although in different siblings). Of the variants, 65 have ccr_per $> 90\%$, 883 have a ccr_per between 1% and 90%, 2786 have ccr_per $= 0\%$, and the remaining 1571 variants did not occur in a CCR region. The variants were divided into deciles based on the reported allele frequency in gnomAD. Cut-offs for the quantiles are in Table 1. This information will be used later to align simulation data to observed ASC counts of variants (Table S2).

Decile	Minor allele frequency threshold
10%	4.88204×10^{-6}
20%	9.70400×10^{-6}
30%	1.92230×10^{-5}
40%	2.91664×10^{-5}
50%	5.10495×10^{-5}
60%	8.36136×10^{-5}
70%	1.44275×10^{-4}
80%	2.49586×10^{-4}
90%	4.46125×10^{-4}
100%	1.00000×10^{-3}

Table S2. Minor allele frequency cut-offs for the gnomAD frequency deciles observed in the ASC data. Variants without a reported gnomAD frequency were excluded from this summary.

We set the number of siblings who can carry *de novo* and/or inherited variants to $N = 2,179$. The number of carriers of *de novo* PTV variants for a gene and the PTV variants that they carry is determined using the following algorithm: (1) randomly determine the number of carriers, m, using a Poisson with $\lambda = 2 \times N \times \mu_i$, where $\mu_i$is the mutation rate to PTV for the gene; (2) randomly sample, with replacement, m PTVs variants from the gnomAD community of PTV variants of the gene; and (3) tabulate which and how often the PTV variants were selected. A rare inherited PTV is defined as a variant that occurs in the child and in at least one of the parents. The PTV cannot occur as a homozygote in any member of the 2,179 trios. The number of carriers of an inherited PTV, h, was simulated using the following algorithm: (1) Simulate genotypes for the 2,179 fathers and 2,179 mothers using a binomial distribution with size = 2 and p = gnomAD minor allele frequency. (2) Exit with $h = NA$ if there is at least one father or mother who are homozygous. (3) Generate the genotype of the children by randomly choosing which allele is transmitted from father and mother using a Bernoulli distribution with $p = 0.5$. (4) Exit with $h = NA$ when there is at least one child with a minor allele homozygous genotype. Return h, the number of children that are heterozygous.

Using these algorithms for 272,929 PTVs in 14,447 genes and for $N = 2,179$ trios yielded ≈ 185 *de novo* PTV and $\approx 15.6K$ inherited PTV occurring in ≈ 7300 genes (over multiple trials). The number of de novo PTV is like that observed in the ASC data, while the number of inherited PTV exceeded that observed in the ASC data. Explanations for this could be a deeper sequencing depth in gnomAD versus ASC, a more restrictive calling algorithm for ASC, different ancestry composition in the two datasets, among others. Closer inspection of the simulated data revealed that approximately ≈ 600 inherited PTVs with $1\% \leq$ ccr_per $< 90\%$, and ≈ 50 with ccr_per $\geq 90\%$. These are about half of what was present in the ASC data.

Therefore, we constructed a down sampling algorithm: (1) Randomly draw a number between 5000 and 5500 to determine the number of PTV required (M). The number of PTV in each minor allele decile is then $M/10$. (2) Accept all de novo PTV, determine their decile bins. (3) Accept the inherited PTV with *ccr_per* ≥ 1, determine their decile bins. (4) Adjust the number of PTV needed for each decile bin by the number of PTVs assigned to the bin in step (2) and (3). (5) The sum of the adjusted number is the total number of inherited PTV with ccr_per < 1 or missing needed to fill the bins (k). (6) Determine the decile bin for inherited PTV with ccr_per < 1 or missing. (7) Calculate the ratio of the number of PTV needed from each bin (step 4) divided by the number available (step 6); (8) Randomly draw k PTV, without replacement, from the inherited PTV with ccr_per < 1 or missing, using the weights from step 7.

Supplementary Material C : Results from analyses of ASC data

We present genes additionally identified by $TADA_{RD}$ compared with $TADA_{CC}$, using a q-value cutoff of 0.05. A total 23 genes are additionally and consistently identified by both *ClassDn* methods (RUSBoost and Underbagging). The complete results for all genes considered are provided in "Supplementary Material C_result.xlsx"

Of 18 genes discovered exclusively by Underbagging, 11 of 18 have some evidence for association ($FDR < 0.30$) in (Fu et al., 2022): *LZTS3, YLPM1, TBR1, SMARCC2, TCF12, NR3C2, NACC1, WDFY3, HECTD4, BRSK2, C20orf112*. Four of these 11 show $FDR < 0.05$ association: *SMARCC2, NACC1, HECTD4, BRSK2*. Mutations in two other genes are known to increase the risk of developmental disabilities, including ASD or intellectual disabilities: *POLR3B, SATB1*. Of the 5 remaining genes, *PDS5B, LRP1, CRP, NUMA1, PCLO*, only two have some other source of evidence for association: *PDS5B* shows overtransmission of PTVs (7 transmitted, none not) and damaging missense variants (2 transmitted, none not); while, for *LRP1*, the literature has some support for association of this gene with ASD and schizophrenia (Torrico et al., 2019). Given these results, a conservative approach would be to use multiple classifiers to identify likely de novo variants.

Table S3 A list of 23 genes additionally discovered by $TADA_{RD}$ compared to $TADA_{CC}$, using a q-value cutoff of 0.05. Only genes commonly identified by both *ClassDn* methods (RUSBoost and Underbagging) are included.

Gene	De novo	Inherited	Case	Likely de novo		q-value		
				rusboost	underbagging	$TADA_{CC}$	$TADA_{RD}$	
							rusboost	underbagging
CREBBP	1	0	2	2	2	0.741	0.036	0.038
MYCBP2	0	0	5	5	5	0.848	0.001	0.001
NFIX	0	1	2	2	2	0.592	0.019	0.021
ZMYND11	1	0	2	2	2	0.157	0.007	0.007
SPEN	2	0	1	1	1	0.191	0.034	0.037
GOLGA5	1	1	2	2	2	0.673	0.015	0.019
MEF2C	0	1	4	3	3	0.501	0.003	0.003
TNRC6B	2	1	3	3	3	0.067	0.000	0.000
EP300	1	1	2	2	2	0.743	0.039	0.041
MIB1	0	18	8	7	8	0.906	0.000	0.000
RAI1	1	0	3	3	3	0.531	0.007	0.007
USP5	0	0	2	2	2	0.680	0.043	0.045
SPTBN1	1	0	2	2	2	0.575	0.021	0.024
IL1R2	0	6	8	4	4	0.899	0.000	0.000
THRB	1	0	1	1	1	0.238	0.048	0.050
TRIP12	2	0	1	1	1	0.111	0.010	0.012
AUTS2	0	2	2	2	2	0.659	0.045	0.047
PPM1D	1	2	3	3	3	0.863	0.003	0.003
TANC2	2	1	2	2	2	0.101	0.002	0.002
UNC119B	0	1	3	3	3	0.564	0.002	0.001
DYNC1H1	1	1	8	4	7	0.098	0.005	0.000
QRICH1	1	0	2	2	2	0.181	0.008	0.008
DLL1	1	0	1	1	1	0.199	0.038	0.040

1. (number of likely inherited variants)=Case-(number of likely de novo variants)
2. A total of 18 genes are discovered exclusively by Underbagging type of *ClassDn*, excluding those identified by $TADA_{CC}$; *POLR3B, PDS5B, LZTS3, YLPM1, LRP1, CRP, TBR1, NUMA1, SMARCC2, TCF12, NR3C2, NACC1, WDFY3, HECTD4, BRSK2, SATB1, PCLO, C20orf112*.
